# Commentary: Bilateral risk-reducing mastectomy is the safest strategy in *BRCA1* carriers

**DOI:** 10.3389/fpsyg.2017.00121

**Published:** 2017-01-31

**Authors:** Rachael Glassey, Christobel Saunders, Sarah J. Hardcastle

**Affiliations:** ^1^Faculty of Medicine, School of Surgery, Dentistry and Health Sciences, University of Western AustraliaPerth, WA, Australia; ^2^Health Psychology and Behavioural Medicine Research Group, Faculty of Health Sciences, School of Psychology and Speech Pathology, Curtin UniversityPerth, WA, Australia

**Keywords:** familial cancer, bilateral prophylactic mastectomy, risk management, counseling, psychological support

Pilgrim and Pain (2014) suggest the best option for BRCA1 carriers is to undergo a bilateral prophylactic mastectomy (BPM). Pilgrim and colleagues suggest that a BPM combined with salpingo-oophorectomy at 25 years old is the best option to minimize risk of developing breast cancer (BC) and ovarian cancer. Undergoing BPM may be the safest option to reduce incidence and mortality, however a diagnosis of a BRCA mutation does not confer a 100% risk and BPM is not the only risk management option. The risk (to 70 years) of developing BC for a BRCA1 carrier is estimated to be about 60% (Mavaddat et al., 2013) and it has been suggested women with BRCA mutations may have up to 80% lifetime risk (National Cancer Institute, 2013). We contend BPM it is not necessarily the best option for all women, especially at such a young age and without a process of fully informed and supported decision-making. The purpose of the current paper is to highlight that health professional recommendations to undergo a BPM may lead to patient regret and clinician blame. Our commentary highlights the consequences of undergoing a BPM without a process of fully informed decision-making, including potential emotional and psychological complications associated with body image and sexuality. We suggest health professionals should provide patients with all options, including screening, medical risk reduction (e.g., tamoxifen) and BPM. We propose all women considering BPM should be referred to an experienced counselor or psychologist to discuss their options, and ensure these individuals are making an informed decision and not one driven by fear or based on the perceived bias of one health professional or other individual, such as anecdotal advice from well-meaning friends or family.

Women with a BRCA mutation are often extremely fearful about developing BC (Dean, 2016). As such, many are driven to undergo BPM out of fear without considering the consequences (Frost et al., 2000). Furthermore, there is evidence to suggest that discussions initiated by a health professional to opt for BPM may lead to regret post-surgery in some women. For example, Payne et al. (2000) found that initiation of discussion about BPM could influence women's decision-making. They found that those who had regrets about their surgery reported the discussion about BPM was initiated by a health practitioner. They concluded that a practitioner-initiated discussion about undergoing BPM predicted regret. More recently, there are reports of litigation on the basis that women were ill-informed of treatment options and left with complications following BPM. Early in 2016 an Australian newspaper reported a story of two women suing a hospital following BPM on the basis that health professionals did not fully explain treatment options to them (Cavazzini and App, 2016). If health professionals encourage BPM simply based on statistical risk reduction and without taking into account other options and potential consequences of surgery, it may increase psychological issues post-surgery and lead to women blaming health professionals if they are unhappy with the outcome or feel they were not fully informed of other options. Regret may be more common if the solution to a proven BRCA mutation is practitioners recommending BPM and women feeling rushed into surgical options, primarily out of fear, and without a process of informed and supported decision-making (Taylor and Tischkowitz, 2014). Health professionals should discuss with women of all their options in an unbiased fashion, which includes BPM as one option. Women who wish to consider the irreversible procedure of BPM should then be referred by their practitioner to a psychologist to discuss these options, including deferring a decision to a later date, especially if women are as young as 25 years old.

Statistically BPM may be the best option to reduce the risk of developing BC. Although the risk is not reduced to zero, BPM can reduce the relative risk that BC will develop by over 90% (Hartmann et al., 1999; Rebbeck et al., 2004). This is important to highlight since there may be the perception amongst patients that BPM reduces the cancer risk to zero, which is not true. The decision to undergo BPM is a personal decision and is often fraught with emotional, psychological and physical complications (den Heijer et al., 2012; Hallowell et al., 2012). The decision needs to be informed and made by the individual (Taylor and Tischkowitz, 2014). A decision to undergo BPM needs to take into account factors such as having children, breast feeding, quality of life and intimacy. A woman's perception of her body can change as a result of BPM, and this can contribute to women feeling less feminine. Women often report their reconstructed breasts look and feel unnatural which makes them feel less attractive and impacts their sex life (Altschuler et al., 2008; Brandberg et al., 2008). Poor cosmetic outcomes, complications from surgery and/or reconstruction are not uncommon and often associated with greater psychological distress (Bebbington Hatcher and Fallowfield, 2003).

For these reasons we suggest that there is a need to provide counseling/psychological consultation to BRCA positive women who are considering BPM. This approach is less likely to lead to practitioner blame and will ensure women are fully informed about the best course of action for them before deciding and undergoing BPM. Patenaude et al. (2008) demonstrated support for psychological consultation before BPM by an experienced and knowledgeable practitioner. Women in their study suggested it would be helpful to anticipate what they might feel after undergoing surgery. An experienced counselor/psychologist is equipped to discuss psychological and emotional implications of a BPM to ensure women have explored all the options and are able to make an informed decision. Women may often not be able or willing to understand the ramifications of this surgery because they are so driven by fear. Emphasis on ensuring women understand the realities of BPM, and making sure they are ready for such surgery, will likely lead to more satisfaction and less psychological adjustment issues following surgery.

In summary, women with BRCA mutations should be fully informed and supported in their decisions around treatment both now and into the future, including understanding both benefits and potential complications of all treatment options. Women who wish to consider BPM should be referred for counseling and psychological support to ensure they understand their options and the implications of undergoing BPM.

## Author contributions

RG and SH conceived the ideas presented in the article. RG drafted the article. Both SH and CS assisted in drafting and refining the article prior to submission.

### Conflict of interest statement

The authors declare that the research was conducted in the absence of any commercial or financial relationships that could be construed as a potential conflict of interest.

## References

[B1] Altschuler A. Nekhlyudov L. Rolnick S. J. Greene S. M. Elmore J. G. West C. N. (2008). Positive, negative, and disparate—women's differing long-term psychosocial experiences of bilateral or contralateral prophylactic mastectomy. Breast J. 14, 25–32. 10.1111/j.1524-4741.2007.00521.x 18186862

[B2] Bebbington Hatcher M. Fallowfield L. (2003). A qualitative study looking at the psychosocial implications of bilateral prophylactic mastectomy. Breast 12, 1–9. 10.1016/S0960-9776(02)00135-2 14659349

[B3] Brandberg Y. Sandelin K. Erikson S. Jurell G. Liljegren A. Lindblom A. (2008). Psychological reactions, quality of life, and body image after bilateral prophylactic mastectomy in women at high risk for breast cancer: a prospective 1-year follow-up study. J. Clin. Oncol. 26, 3943–3949. 10.1200/JCO.2007.13.9568 18711183

[B4] Cavazzini M. App (2016). Prophylactic Mastectomy Ends in Court. Chatswood, DC: Australian Doctor.

[B5] Dean M. (2016). “It's not if I get cancer, it's when I get cancer”: BRCA-positive patients' (un)certain health experiences regarding hereditary breast and ovarian cancer risk. Soc. Sci. Med. 163, 21–27. 10.1016/j.socscimed.2016.06.039 27376595

[B6] den Heijer M. Seynaeve C. Timman R. Duivenvoorden H. J. Vanheusden K. Tilanus-Linthorst M.. (2012). Body image and psychological distress after prophylactic mastectomy and breast reconstruction in genetically predisposed women: a prospective long-term follow-up study. Eur. J. Cancer 48, 1263–1268. 10.1016/j.ejca.2011.10.020 22105017

[B7] Frost M. H. Schaid D. J. Sellers T. A. Slezak J. M. Arnold P. G. Woods J. E. (2000). Long-term satisfaction and psychological and social function following bilateral prophylactic mastectomy. JAMA 284, 319–324. 10.1001/jama.284.3.319 10891963

[B8] Hallowell N. Baylock B. Heiniger L. Butow P. N. Patel D. Meiser B. (2012). Looking different, feeling different: women's reactions to risk-reducing breast and ovarian surgery. Fam. Cancer 11, 215–224. 10.1007/s10689-011-9504-4 22198037

[B9] Hartmann L. C. Schaid D. J. Woods J. E. Crotty T. P. Myers J. L. Arnold P. (1999). Efficacy of bilateral prophylactic mastectomy in women with a family history of breast cancer. New Engl. J. Med. 340, 77–84. 9887158 10.1056/NEJM199901143400201

[B10] Mavaddat N. Peock S. Frost D. Ellis S. Platte R. Fineberg E.. (2013). Cancer risks for BRCA1 and BRCA2 mutation carriers: results from prospective analysis of EMBRACE. J. Natl. Cancer Inst. 105, 812–822. 10.1093/jnci/djt095 23628597

[B11] National Cancer Institute (2013). BRCA1 and BRCA2: Cancer Risk and Genetic Testing. Available online at: http://www.cancer.gov/cancertopics/factsheet/Risk/BRCA

[B12] Patenaude A. F. Orozco S. Li X. Kaelin C. M. Gadd M. Matory Y. (2008). Support needs and acceptability of psychological and peer consultation: attitudes of 108 women who had undergone or were considering prophylactic mastectomy. Psychooncology 17, 831–843. 10.1002/pon.1279 18636423 PMC4133134

[B13] Payne D. K. Biggs C. Tran K. N. Borgen P. I. Massie M. J. (2000). Women's regrets after bilateral prophylactic mastectomy. Ann. Surg. Oncol. 7, 150–154. 10.1007/s10434-000-0150-6 10761795

[B14] Pilgrim S. Pain S. (2014). Bilateral risk-reducing mastectomy is the safest strategy in *BRCA1* carriers. Eur. J. Surg. Oncol. 40, 670–672. 10.1016/j.ejso.2014.02.218 24612652

[B15] Rebbeck T. R. Friebel T. Lynch H. T. Neuhausen S. L. van't Veer L. Garber J. E. (2004). Bilateral prophylactic mastectomy reduces breast cancer risk in BRCA1 and BRCA2 mutation carriers: the PROSE Study Group. J. Clin. Oncol. 22, 1055–1062. 10.1200/JCO.2004.04.188 14981104

[B16] Taylor A. Tischkowitz M. (2014). Informed decision-making is the key in women at high risk of breast cancer. Eur. J. Surg. Oncol. 6, 667–669. 10.1016/j.ejso.2014.02.219 24655800

